# Educational

**Published:** 1885-07

**Authors:** 


### Educational.

We would call especial attention to the following announcement. It is very desirable that all the reputable dental colleges of this country be represented in this Association. When this is attained, if the education of those who enter the profession hereafter are not up to a proper standard, it will not be a difficult matter to decide upon whom rests the responsibility. The control of our professional education rests, in a large measure, upon the colleges.

If they unite and cöoperate their influence will be irresistible.

CINCINNATI, May 15, 1885.

The Annual Meeting of the National Association of Dental Faculties will be held at the Sherman House, in Chicago, at 11 o'clock A. M., July 31st.

By order of the Executive Committee.

H. A SMITH, Secretary. C. N. PEIRCE, President.

ARTICLE VII OF THE CONSTITUTION.

*Membership:* Any reputable dental college may be represented in this body upon submitting to the executive committee satisfactory credentials, signing the constitution, conforming to the rules and regulations of this body, and paying such assessments as may be made.

